# Improvement of silage characteristics of *Lactobacillus salivarius* HMC4 and improvement of silage quality of king grass

**DOI:** 10.3389/fmicb.2024.1468577

**Published:** 2024-12-11

**Authors:** Jinsong Yang, Songsong Zhao, Wenbo Zhi, Tianyu Lu, Huahua Qiao, Wei Liu, Ying Dou, Haisheng Tan, Hanlin Zhou

**Affiliations:** ^1^College of Food Science and Engineering, Hainan University, Haikou, Hainan, China; ^2^College of Materials Science and Engineering, Hainan University, Haikou, Hainan, China; ^3^Zhanjiang Experimental Station, Chinese Academy of Tropical Agricultural Sciences, Zhanjiang, China; ^4^Key Laboratory of Ministry of Agriculture and Rural Affairs for Germplasm Resources Conservation and Utilization of Cassava, Key Laboratory of Ministry of Agriculture and Rural Affairs for Crop Gene Resources and Germplasm Enhancement in Southern China, Tropical Crops Genetic Resources Institute, Chinese Academy of Tropical Agricultural Sciences, Danzhou, China

**Keywords:** protoplast fusion, silage, Lactobacillus, high-throughput sequencing, microbial communities

## Abstract

The effect of HMC4 produced by protoplast fusion on silage was studied. The silage formula was composed of heterozygote HMC4 (Group C), parent Lactobacillus (Group A) and a combination of two parents (Group B). The fermentation quality and microbial composition of each batch of silage were evaluated. The results showed that the propionic acid concentration in group C was the lowest, but the organic acid content in feed was significantly increased. Groups B and C had slightly lower crude fiber levels than group A, while groups A and C had higher levels of soluble sugars. The dynamic observation of C group showed that the nutrient composition of feed changed gradually with the extension of silage period. High-throughput sequencing revealed fluctuations in microbial composition before and after silage. Over time, Lactobacillus became the dominant strain and its numbers steadily increased.

## 1 Introduction

King grass is considered a premium type of forage grass suitable for silage or fresh feed. King grass thrives in tropical and subtropical climates characterized by warmth and humidity, but it is susceptible to extreme cold and drought (Chen et al., 2022). Additionally, king grass boasts high yields and excellent nutritional qualities. During summer, harvesting can occur every 30–40 days, while in other seasons, it’s typically every 60 days. The general yield exceeds 10 tons per mu, with high yields surpassing 20 tons (Long et al., 2022). Harvesting frequency ranges from five to eight times annually. Furthermore, king grass serves as a rich source of lignocellulosic materials suitable for ethanol production (Cardona et al., 2016). Although king grass is a significant feed source for ruminants, its availability is low during summer and excessive during winter (Li et al., 2014). The standard method to mitigate this issue effectively involves ensiling king grass abundantly available during summer to achieve long-term preservation objectives (Long et al., 2022).

Silage, a significant component of ruminant diets, typically comprises fermented plant material stored in a sealed environment with high moisture content. The rapid development of animal husbandry is increasing the demand for silage (Yang et al., 2016). Anaerobic conditions are essential for silage fermentation as they inhibit the activity of aerobic microorganisms like bacteria, yeasts, and molds. Additionally, various microorganisms inhabit silage, among which Lactobacillus play a crucial role. The growth of Lactobacillus effectively suppresses the proliferation of other microorganisms (Liu et al., 2014). Adding various strains of Lactobacillus, along with other microbial agents, significantly influences the microbial composition of silage (Wang et al., 2006). Even in this condition, cells and aerobic microbes continue to respire, absorbing oxygen and generating heat. Optimal heat levels can actually promote the growth of Lactobacillus. During the mid-stage of silage, the feed experienced anaerobic conditions as oxygen was consumed in carbon dioxide production. Consequently, Lactobacillus fermented vigorously, producing lactic acid. The rapid decrease in pH, due to increased organic acid content, suppressed aerobic microbes in the silage’s anaerobic and acidic conditions (Ennahar et al., 2003; Yang et al., 2010). Following an extended lactic acid fermentation period, the silage reached stability. At this stage, both the physical and chemical properties of the silage stabilized, with a pH below 4.0 (Du et al., 2022). Pasture grass in natural settings often lacks sufficient Lactobacillus for ideal silage fermentation. Lactobacillus are commonly added to silage to enhance mutual fermentation and accelerate lactic acid fermentation, along with *Lactococcus lactis* found naturally on forage grass.

A protoplast is a piece of cell tissue that has lost its cell wall and contains all of its cytoplasm as well as organelles, which are encased in the cell membrane. Protoplast fusion technology is the process of fusing two protoplasts together to create a hybrid cell in a hypertonic environment using physical, chemical, or biological means. Protoplast preparation, fusion cell production, fusion cell wall regeneration, and fusion cell screening are all steps in the overall process of protoplast fusion. Protoplast fusion is a potent method for creating target strains because it can blend the DNA of several organisms and filter out the strains with certain properties based on requirements. By overcoming the species isolation of conventional hybridization systems and attaining remote hybridization, protoplast fusion of parents is more widespread and may be employed to achieve interspecific or even intergeneric hybridization (Verma et al., 2008). The experimental equipment requirements for this technique are modest, and it is simpler to run. It may be bred by straightforward physiological or chemical perturbations in accordance with the phenotypic features of the parents, and it does not require sophisticated genetic study of the genomes of both parents (Davey et al., 2005; Savitha et al., 2010). Since whole genes with longer lengths can have more possibilities for recombination and more gene combinations than gene fragments, protoplast fusion is a more random process that unites all of the genetic material in a single cell (Hospet et al., 2023). The target of fusion has steadily shifted from basic animal cells to plant cells as protoplast fusion technology has advanced and developed, and there is currently a reasonably developed body of study on microbes. Yang employed protoplast fusion technique to create hybrids with greater alcohol tolerance and fermentation performance than the parents, hence increasing the taste attributes of rice wine (Yang et al., 2018). They acquired outstanding parents using ethanol domestication and UV mutagenesis. In the Ge study, the high temperature resistance of the strain was improved by combining *Bacillus subtilis* and *Lactobacillus rhamnosus* (Ge et al., 2021). Yu created potato seeds resistant to bacterial wilt by crossing the parents of potatoes and eggplant (Yu et al., 2013). Studies have shown that the fusion of microbial strains from Bordetella sp. and Microbacterium sp., both resistant to various pesticide environments, has resulted in a hybrid strain capable of simultaneously degrading two pesticides with an efficiency of up to 70% (Xue et al., 2022). These studies have shown that protoplast fusion provides a fresh solution to issues related to strain breeding, biological control, and suppression of yeast breakdown.

The modified HMC4 heterozygote of *Lactobacillus salivarius*, the parent Lactobacillus, and a mixed bacterial solution of two parents were added to king grass in this experiment. The levels of crude protein, crude fiber, and other nutrients were determined, together with the physicochemical parameters, in order to compare and analyze the effects of fusion on the quality of silage. The microbial composition of every silage group was investigated using high-throughput sequencing technology. The effects of the HMC4 on the microflora and the parents on the microbiological makeup of the silage were investigated using both direct and dynamic bagging.

## 2 Materials and methods

### 2.1 Heterozygote HMC4

The heterozygote HMC4 obtained by the fusion of Lactobacillus sialiae HSRF5 (parent A) and *Bacillus cereus* HCJ4 (parent B) using protoplasts, HSRF5 (parent A) entry number is AB559701.1, HCJ4 (parent B) entry number is MF977311.1, and heterozygote HMC4 entry number is MH473235.1.

### 2.2 King grass silage modulation

Fresh king grass was harvested from the experimental base of the Institute of Tropical Crop Variety Resources at the Chinese Academy of Tropical Agricultural Sciences and dried to regulate moisture content to 65–70%. Following the addition of forage grass and bacterial solutions to the silage bag, groups were organized according to the table below and sealed using vacuum packing equipment. Each group was replicated three times. Store the sealed silage at room temperature and protect it from light exposure. The silage bags were opened on the 50th day post-silage, except for the raw material and hybrid groups. The hybrid group underwent dynamic opening for follow-up testing on the 3rd, 7th, 14th, 28th, and 50th days post-silage.

### 2.3 Determination of fermentation quality of silage

(1)  Determination of fermentation quality of silage

Once the silage is completed, mix it with distilled water at a ratio of 1:9, grind the mixture until the feed tissue is completely broken down, strain the resulting juice through gauze, and then filter it using filter paper. The pH of the filtered juice was measured using a pH meter, and any remaining juice was refrigerated at −20°C. Paper envelopes containing a specific grade of silage were weighed until a constant bulk mass was achieved, then cooled to room temperature in a desiccator, and subsequently placed in an oven set at 105°C for two-hour intervals. Water content is calculated as the ratio of the mass lost during heating to the original mass, with the feed’s weight remaining constant.

(2)  Determination of crude fiber, neutral detergent fiber and acid detergent fiber content

After consecutive treatment with hot sulfuric acid and hot alkali, the residues are acid detergent fiber, neutral detergent fiber, and crude fiber. In this study, the feed was dried to a constant weight, then finely ground to 100 meshes using a Fibertec 2010&M6 fiber analyzer to determine the levels of crude fiber, neutral detergent fiber, and acid detergent fiber.

(3)  Determination of crude protein in silage

In this study, nitrogen oxides produced during protein combustion are reduced to nitrogen, and the protein content is quantified by measuring nitrogen levels using the combustion method commonly employed for protein determination in samples. Test samples were dehydrated, wrapped in tin foil (100 mg each), and subjected to protein content analysis using Dumas nitrogen determination equipment. Aspartic acid served as the standard, and the instrument operated at flow rates of 700 mL/min for CO_2_ and 200 mL/min for O_2_.

(4)  Content of organic acids in silage

The concentration of organic acids in silage filtrate was determined by high performance liquid chromatography. Similar to lactic acid, a series of lactate standard solutions are prepared with reference to GB/T 23877–2009, with all organic acids in the liquid phase. According to the peak area and retention time of acetic acid, propionic acid and butyric acid standard solutions, the standard curves of each organic acid were generated. The concentration of organic acids in feed was determined by comparing feed spectrum with standard curve. The standard curve for organic acids is:

Acetic acid standard curve: *y* = 692.3593x + 3.07657, *R*^2^ = 0.999.

Propionic acid standard curve: *y* = 652.76607x + 2.40482, *R*^2^ = 0.999.

Butyric acid standard curve: *y* = 344.83591x-0.86155, *R*^2^ = 0.999.

(5)  Determination of soluble sugars

The anthrone-sulfuric acid method, as per group standard T/HXCY-2021, was employed to determine the soluble sugar content in silage.

①  Determination of standard curve of sucrose

**Figure 1:**
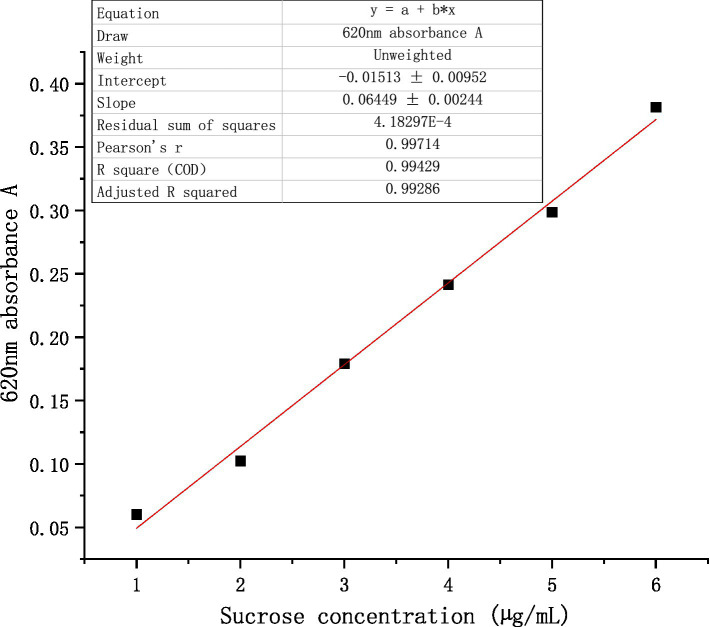
Standard curve of sucrose.

Various sucrose standard solutions ranging from 1 to 6 μg/mL were prepared. Following the addition of a 6.00 mL anthracenone-sulfuric acid solution, the mixture was subjected to a two-minute ice water bath followed by a five-minute boiling water bath. Once the reaction was complete, the absorbance at 620 nm was measured, and the standard curve was plotted and recorded after cooling to room temperature under running water. The following is the standard curve of absorbance y as a function of sucrose concentration x: y = 0.06449x + 0.01513 (Figure 1).

②  Determination and calculation of soluble sugars in samples

Mix 0.5 g of dry feed with 7 mL of 80% ethanol solution, cover, and incubate in a water bath at 80°C for 30 min. After cooling, adjust the volume to 50 mL, then measure the absorbance at 620 nm by sampling the supernatant. The mass of soluble sugar in the test solution is determined using the standard curve, and the soluble sugar content of the sample is calculated using the following formula.


$$X=\frac{C\times 50\times n}{2\times W}\times 100\%$$

The formula involves the following terms: *n* = dilution multiple, 2 = measure absorbance dilution volume, W = sample mass (g), C = mass of soluble sugar derived by standard curve conversion (*μ* g), X = soluble carbohydrate content in the sample (g/kg), and 50 = constant volume (g).

### 2.4 Analysis of microbial composition of silage

Three to five-gram silage samples were collected at the end of each experimental group and stored at −80°C until the completion of silage fermentation in all groups. DNA extraction from the feed of each experimental group was performed using the MOBIO PowerSoil^®^ DNA Isolation Kit, followed by PCR amplification using the V3-V4 universal primer set (341F:ACTCCTACGGGAGGCAGCA; 806R:GGACTACHVGGGTWTCTAAT). The amplified products were subsequently retrieved and sent to Guangdong Mege Genome Technology Co., Ltd. for high-throughput sequencing.

### 2.5 Data processing

Each group underwent three experimental iterations. The function curve was constructed using Origin software, and data were analyzed using SPSS software. The output result included the mean ± standard deviation. After rounding the data to two decimal places (*p* < 0.05), a significant change was observed. The strain development tree was constructed using MEGA11. Visuals related to high-throughput sequencing were generated using the Tukey test, Kruskal-Walla test within groups, t-test, Wilcoxon rank test between groups, and R language.

## 3 Results

### 3.1 Analysis of fermentation quality of silage

This study investigated the quality and strain differences in silage between HMC4 and their parents, as well as the influence of HMC4 on the nutritional content and microbial composition of silage, employing both direct and dynamic bag opening techniques (Tables 1, 2). Table 3 presents the results of the analysis of the physical and chemical properties, nutritional value, and organic acidification of the feed.

**Table 1:** Experimental grouping and inoculation amount.

Group	Silage time (day)	Addition amount of bacterial liquid	Forage grass addition	Quantity/bag
Control Check (CK)	0	0	1,000 g	3
Heterozygote (C3)	3	2 mL HMC4	1,000 g	3
Heterozygote (C7)	7	2 mL HMC4	1,000 g	3
Heterozygote (C14)	14	2 mL HMC4	1,000 g	3
Heterozygote (C28)	28	2 mL HMC4	1,000 g	3
Heterozygote (C50)	50	2 mL HMC4	1,000 g	3
Lactobacillus group (A)	50	2 mL parent A	1,000 g	3
Mixed Group (B)	50	1 mL parent A + 1 mL parent B	1,000 g	3
Model group (M)	50	0	1,000 g	3

50 days are allotted for silage. Following the addition of HMC4 to king grass, group A’s bags were left open in silage for 3, 7, 14, 28, and 50 days, in that order.

**Table 2:** PCR amplification conditions for high-throughput sequencing.

PCR reaction reagent	Dosage/specification	PCR amplification program
2x Premix Taq	25 μL	94°C 5 min
Primer-F (10 μM)	1 μL	94°C 30 s
Primer-R (10 Μm)	1 μL	52°C 30 s 30 cycles
DNA	50 ng	72°C 30s
Nuclease-free water	Add to 50 μL	72°C 10 min
PCR instrument	BioRad S1000 (Bio-Rad Laboratory, CA)	4°C Hold

**Table 3:** Analysis of each component of silage.

Group/project	pH	Moisture /%	CP/%	WSC/g/kg	Crude fiber/%	NDF/%	ADF/%	Lactic acid/%	Acetic acid/%	Propionic acid/%	Butyric acid/%
CK (Day 0)	4.51 ± 0.01a	66.46 ± 1.00a	6.75 ± 0.50b	10.36 ± 0.18a	40.23 ± 0.30b	76.22 ± 0.28	56.53 ± 0.25ab	0.32 ± 0.03f	1.01 ± 0.02d	—	—
C3 (HMC4 3 days)	4.35 ± 0.07b	65.14 ± 0.19ab	5.94 ± 0.12 cd	5.08 ± 0.02b	39.68 ± 0.34b	76.08 ± 0.51	57.21 ± 1.13a	0.63 ± 0.03f	1.75 ± 0.45c	—	—
C7 (HMC4 7 days)	4.14 ± 0.11c	63.72 ± 0.79bcd	5.88 ± 0.45 cd	3.03 ± 0.07c	42.27 ± 0.67b	76.67 ± 0.85	57.82 ± 0.81a	2.71 ± 1.46e	1.64 ± 0.25c	—	—
C14 (HMC4 14 days)	4.04 ± 0.10 cd	65.88 ± 1.60a	5.94 ± 0.04 cd	2.88 ± 0.01c	39.81 ± 0.87b	76.43 ± 0.44	55.71 ± 1.79abc	3.82 ± 0.90d	1.90 ± 0.43c	—	—
C28 (HMC4 28 days)	3.89 ± 0.04e	64.44 ± 1.98abc	7.21 ± 0.25a	2.88 ± 0.04c	40.91 ± 0.32b	76.37 ± 1.47	52.85 ± 0.35c	4.83 ± 0.06bcd	2.40 ± 0.03b	0.30 ± 0.03a	0.11 ± 0.01
C28 (HMC4 50 days)	3.86 ± 0.02e	60.89 ± 0.59e	6.26 ± 0.07c	2.52 ± 0.05d	40.51 ± 0.15b	76.13 ± 5.26	53.34 ± 1.84c	6.20 ± 0.48a	3.12 ± 0.01a	0.18 ± 0.19b	0.12 ± 0.00
A (Lactobacillus for 50 days)	3.96 ± 0.02de	62.06 ± 0.95de	6.17 ± 0.03c	2.40 ± 0.03d	42.28 ± 0.89b	75.22 ± 1.28	52.89 ± 2.23c	4.33 ± 0.05 cd	2.54 ± 0.06b	0.28 ± 0.00ab	0.11 ± 0.03
B (mixture for 50 days)	3.94 ± 0.02de	62.73 ± 1.19cde	6.26 ± 0.10c	1.68 ± 0.17e	40.74 ± 0.18b	75.34 ± 0.24	53.69 ± 1.86bc	4.97 ± 0.04bc	2.58 ± 0.04b	0.30 ± 0.01a	0.14 ± 0.05
M (blank for 50 days)	4.04 ± 0.01 cd	65.62 ± 0.55ab	5.52 ± 0.13d	2.48 ± 0.06d	60.95 ± 5.54a	76.17 ± 0.38	54.04 ± 1.73bc	5.49 ± 0.30ab	3.02 ± 0.16a	0.35 ± 0.03a	0.15 ± 0.04

The data in the table is the average ± standard deviation, a,b Values within a row with different superscripts differ significantly at p < 0.05. “—” indicates that it has not been detecte.

Despite a slight decrease, the moisture level of the feed remained above 65% both before and after preparation. However, the water content dropped significantly to approximately 61% after the addition of microbial preparation. The raw material of this silage contains approximately 6.7% crude protein (CP), which decreases after the silage process. Nonetheless, adding a bacterial agent to the preparation process can significantly minimize the loss of crude protein, maintaining the protein content at around 6.2 percent. There was a noticeable decrease in the amount of soluble sugar (WSC) as the silage progressed. Initially, the soluble sugar content was around 10.3 g/kg, which decreased to approximately 2.5 g/kg by the end of the silage process. HMC4 silage had slightly higher soluble sugar content compared to mixed-group silage, which had the lowest amount at around 1.6 g/kg. The crude fiber content of blank silage increased significantly from the raw material to 60%, whereas the bacterial agent-treated silage experimental group successfully mitigated this increase, maintaining the crude fiber level at approximately 40%. The concentration of neutral detergent fiber (NDF) in feed ranged from 75 percent to nearly constant levels after silage. The concentration of acid detergent fiber (ADF) decreased before 14 days of silage, with a gradual reduction observed as the silage period progressed. The ADF content of the blank group remained relatively stable after 28 days of silage, but it decreased significantly from the initial 55 percent to approximately 52 percent. Organic acids are the primary factors influencing pH value changes in silage. The concentrations of lactic acid, acetic acid, propionic acid, and butyric acid were measured in each experimental group. The findings indicated that the primary organic acids in the early stages of silage (0–3 days) were acetic acid and lactic acid. In the intermediate stages of silage (3–14 days), lactic acid concentration rose quickly, but acetic acid content remained relatively constant. Propionic acid and butyric acid were undetectable in any of the experimental groups before the 28-day silage period. However, after 28 days, a small amount of these acids was detected in the feed, and the overall composition of organic acids remained largely consistent, with lactic acid and acetic acid comprising the majority. The ultimate concentrations of propionic acid and butyric acid did not differ significantly between the experimental groups. The heterozygous experimental group had the highest concentrations of lactic acid and acetic acid, at around 6 and 3%, respectively, significantly exceeding those of the other experimental groups. The concentrations of lactic acid and acetic acid in the blank model group were likewise higher, at 5 and 3%, respectively, compared to 4.9 percent and 2.5 percent in the mixed experimental group. Lastly, following silage, the lactic acid and acetic acid concentrations in the Lactobacillus group were considerably lower than those in the blank model group, which were 4.3 percent and 2.5 percent, respectively. Overall, the heterozygote group >blank model group ≥mixed silage group ≥Lactobacillus group was seen in the lactic acid content (Table 3).

### 3.2 Analysis of microorganisms in silage

#### 3.2.1 High-throughput sequencing results

**Figure 2:**
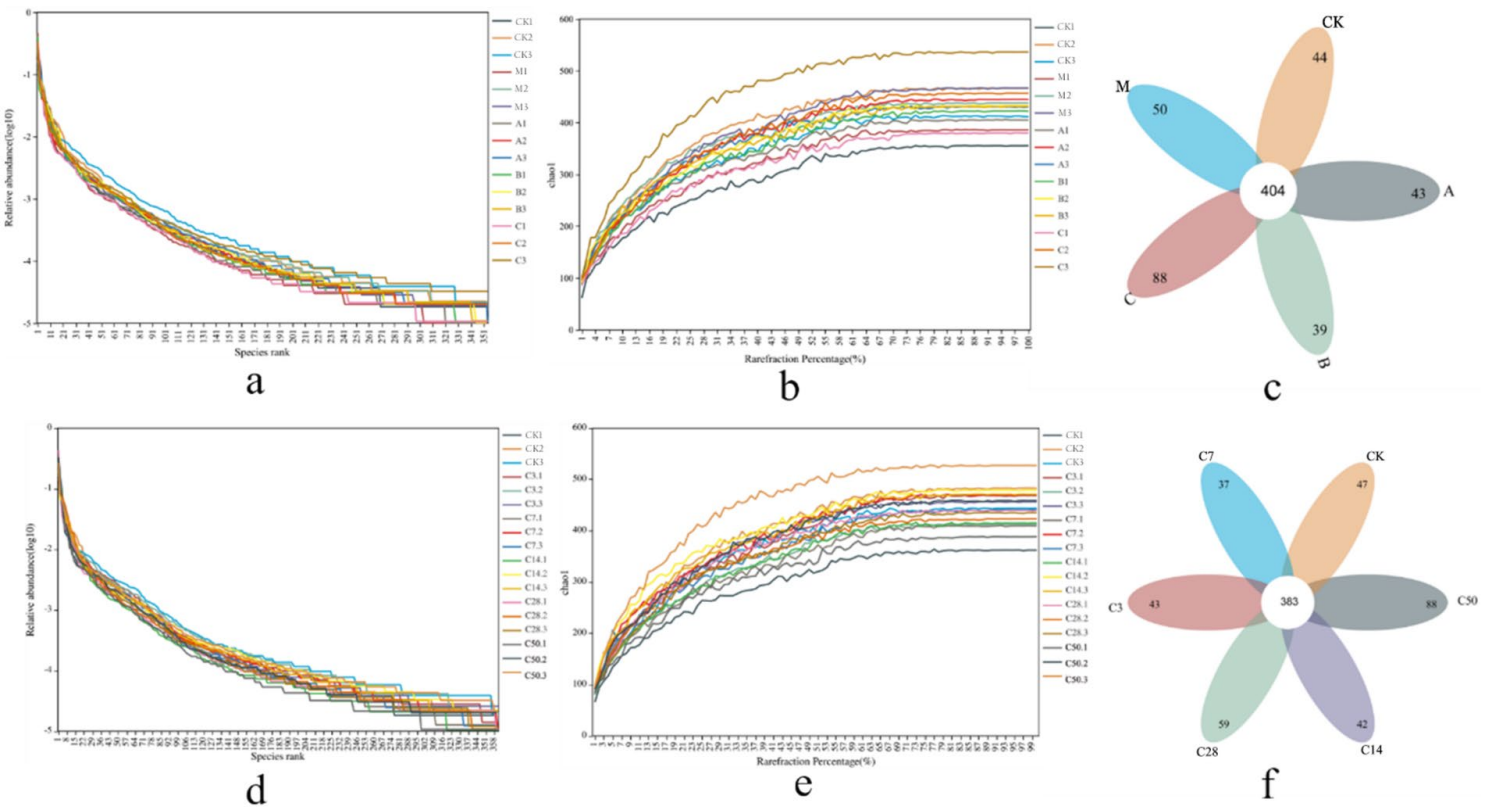
Sequencing assembly results. Panel (a–c) are the abundance curve, dilution curve, and petal diagram after sequence assembly under direct open bag grouping; (d–f) are the abundance curve, dilution curve, and petal diagram under dynamic open bag grouping.

Dilution and abundance curves serve as indicators of sequencing depth and accuracy. A stable curve suggests that sequencing criteria have been achieved (Figures 2a,b,d,e). Sequencing produced 6,452 OUT messages for the two groups (direct bag opening and dynamic bag opening) and 7,966 OUT messages for the other group (1,349,329 and 1,470,230, respectively). As shown in Figure 2c, in the direct bag-opening group, an average of 456 effective OUT messages were detected in each experimental group, among which 404 were common OUT messages. According to the sequence of raw material group, model control group, lactic acid bacteria group, mixed group and hybrid group, there were 44, 50, 43, 39 and 88 independent OUT messages in each silage group. Additionally, Figure 2f illustrated that 383 shared messages were identified among an average of 435 valid OUT messages in each experimental group. The sequence of silage time revealed that each silage group displayed 47, 43, 37, 42, 59, and 88 unique OUT signals.

#### 3.2.2 Microbial diversity analysis

*α*-analysis was conducted to assess the sequencing data. Table 4 shows that the sample’s species richness was assessed using the Chao1 and ACE indexes, and its community diversity was evaluated using the Shannon and Simpson indexes.

**Table 4:** Alpha index of microbial diversity in each experimental group.

Sample name	Chao1	ACE	Shannon	Simpson
CK (Day 0)	429.73 ± 61.55	509.28 ± 68.04	3.40 ± 0.39	0.08 ± 0.03
C3 (HMC4 3 days)	446.07 ± 29.35	535.38 ± 46.56	3.41 ± 0.17	0.08 ± 0.02
C7 (HMC4 7 days)	440.13 ± 30.50	538.07 ± 35.11	3.14 ± 0.20	0.12 ± 0.03
C14 (HMC4 14 days)	455.17 ± 34.85	533.89 ± 30.93	3.20 ± 0.30	0.11 ± 0.04
C28 (HMC4 28 days)	432.63 ± 8.52	499.78 ± 14.66	3.05 ± 0.31	0.13 ± 0.07
C50 (HMC4 50 days)	458.10 ± 69.50	525.39 ± 53.32	3.04 ± 0.33	0.13 ± 0.03
A (Lactobacillus for 50 days)	426.93 ± 20.02	494.39 ± 24.16	3.18 ± 0.15	0.10 ± 0.04
B (mixture for 50 days)	428.90 ± 5.66	501.84 ± 4.98	3.13 ± 0.23	0.12 ± 0.05
M (blank for 50 days)	430.20 ± 41.03	497.75 ± 48.09	2.96 ± 0.36	0.16 ± 0.07

There is no significant difference in the table.

The α-analysis suggests that the α index of each sample group remains relatively stable, indicating that the overall species richness and diversity in the feed environment are not significantly affected by the duration of silage or silage bacteria.

**Figure 3:**
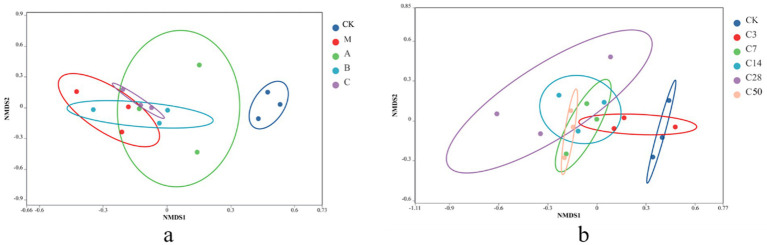
NMDS analysis with different additives (a) and different silage times (b).

Non-metric multidimensional scaling (NMDS) analysis was conducted to examine the samples. In NMDS, samples are represented as points in a multidimensional space based on species information, with the distance between points indicating the relationship between samples. The degree of similarity in the species information between the two samples increases with the distance between the two sample places. Figure 3 depicts that the microbial composition of raw material king grass significantly differs from that of silage. Silage with Lactobacillus addition exhibits a microbial composition more similar to fresh feed compared to direct silage without treatment. The microbial composition of the HMC4 experimental group lies between Lactobacillus and mixed silage. Figure 3b demonstrates the progressive variation in microorganism composition in the feed as the silage duration increases. The control check is scattered on the right, whereas the silage group is predominantly distributed on the left. Even after 3 days of silage, there was overlap in the microbial makeup between the control check and the silage group. After 7 days of silage, the control check and the silage group became completely separated. The microbial makeup of the two groups exhibited significant differences after 50 days and 3 days of silage, respectively.

Overall, the addition of strain supplements to silage did not significantly impact species richness and diversity indices. Nevertheless, with the prolonged silage period, the microorganism composition in the feed underwent progressive changes, with variations depending on the bacterial agent employed.

#### 3.2.3 Species composition analysis

**Figure 4:**
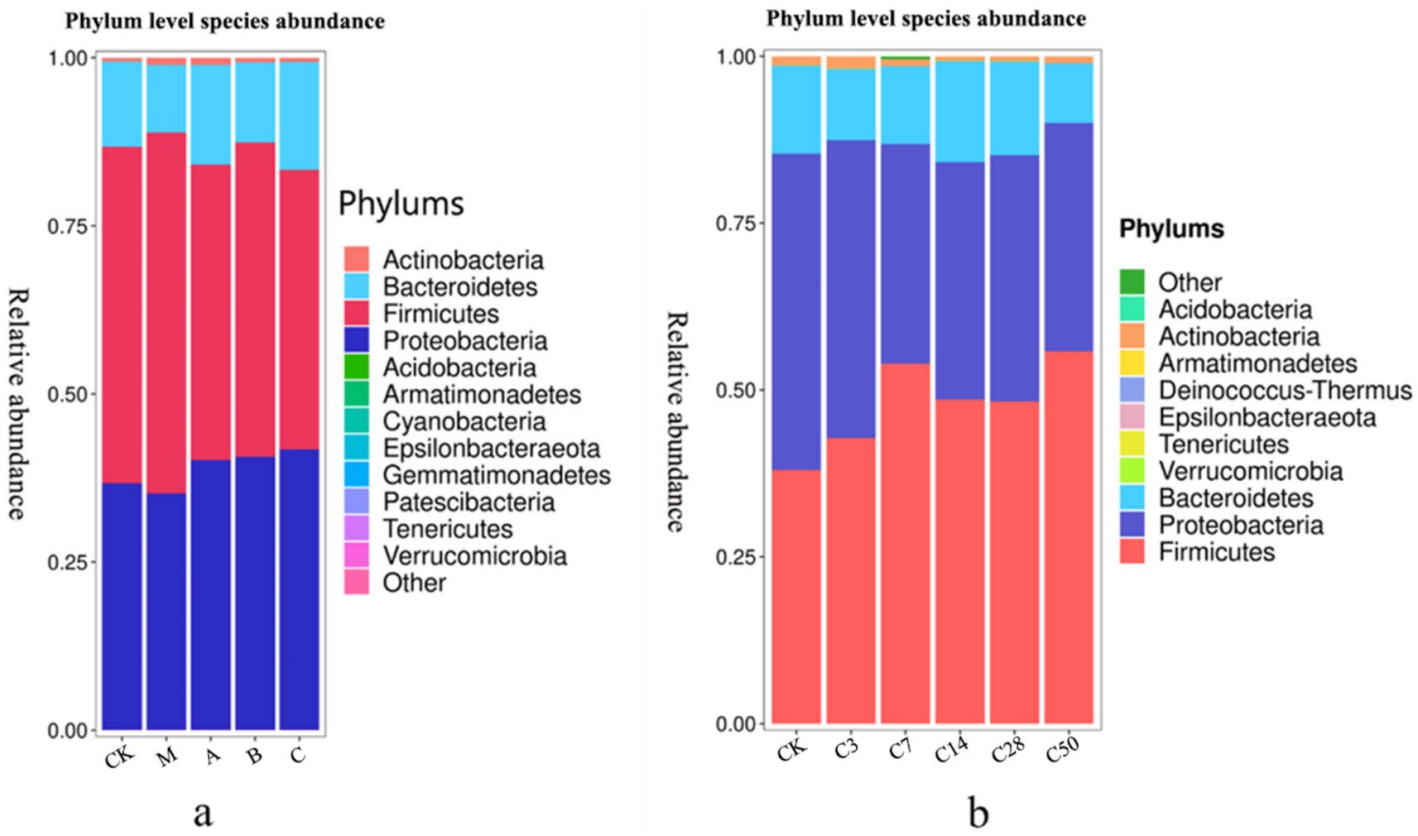
Phylum level species stacking plots for different additives (a) and different silage times (b).

**Figure 5:**
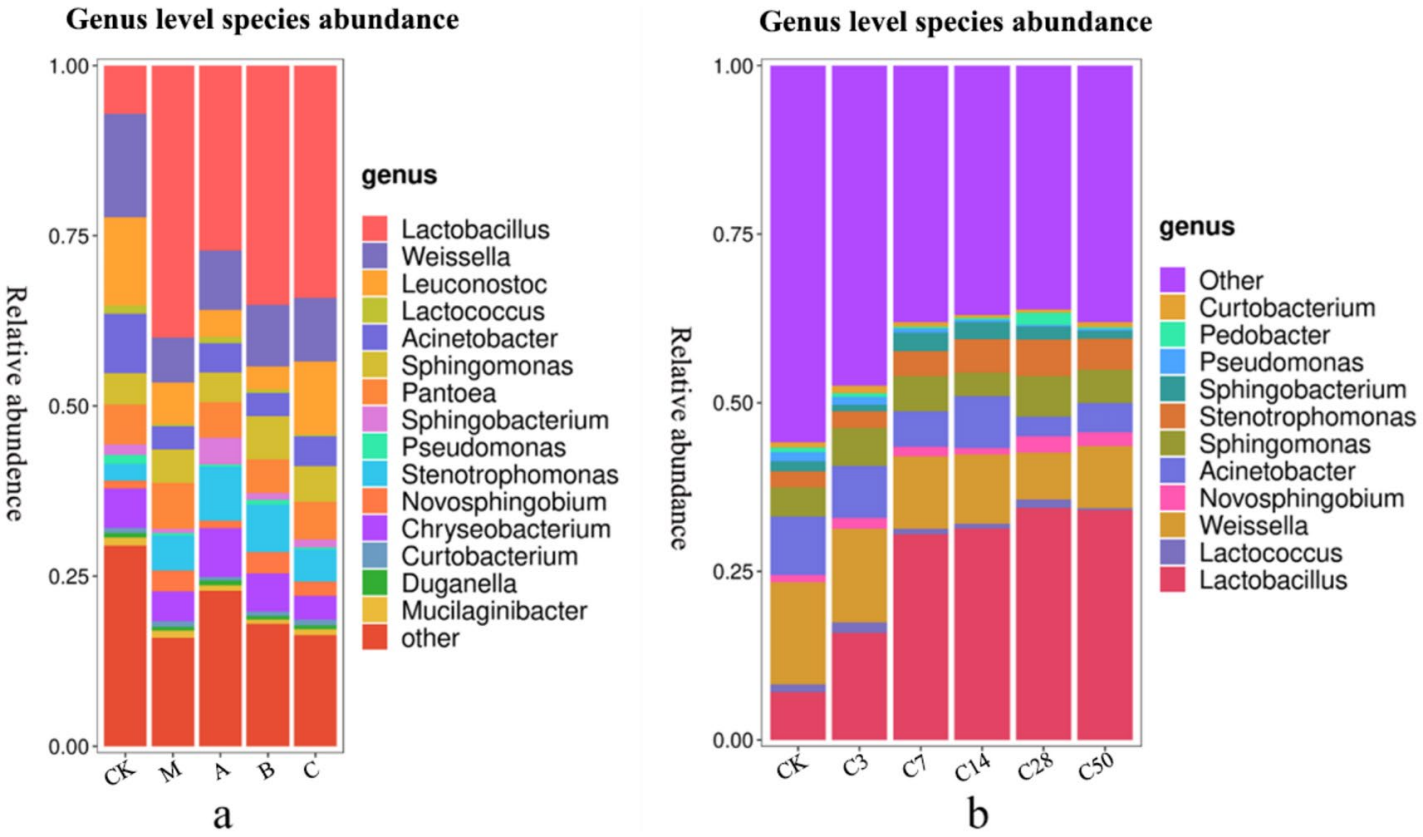
Genus level species stacking plots for different additives (a) and different silage times (b).

Species accumulation Figures 4, 5 show that Streptomyces, Proteus, Bacteroides and actinomyces are the dominant microorganisms with high abundance during silage fermentation. The abundance of these bacteria varies depending on the silage material and fermentation time. The abundance of Streptomyces in raw material group, blank group, lactic acid bacteria group, mixed fermentation group and heterozygote group was 50.04, 53.67, 43.98, 46.72, and 41.64%, respectively.

Proteus was present in 36.67, 35.16, 40.07, 40.06, and 41.65% of the samples, respectively. Actinomycetes were present in 0.56, 1.07, 1.04, 0.68, and 0.63% of the samples, respectively, while Bacteroides were present in 12.69, 10.04, 14.84, 11.95, and 16.03% of the samples, respectively. In feed, the addition of Lactobacillus significantly increased the abundance of Proteus and Pseudomonas, while Phaeophyta was less abundant.

Lactobacillus, Weissiella, Acinetobacter, *Candida albicans*, Sphingosine monomonas, Pantobacter, Flavobacterium, Oligotrophomonas, Neo-sphingosine bacteria, and Sphingomycetes are among the top 10 microbial genera with high abundance at the genus level. Unlike the static bag opening method, dynamic bag opening observed fluctuations in microbe abundance over time, ultimately revealing outcomes consistent with direct bag opening. For example, the initial presence of Pseudomonas in the raw material was approximately 1.34% in the early fermentation stages. However, following silage, this presence steadily declined, and upon bag opening, it was reduced to just 0.26%.

Following silage, a significant decrease in Acinetobacter abundance and a notable increase in Lactobacillus abundance were observed compared to the initial groups (*p* < 0.05). Additionally, the abundance of *Candida albicans* in the mixed fermentation group was significantly higher compared to the original king grass (*p* < 0.042). Moreover, Pseudomonas was significantly less abundant in the heterozygote group (*p* = 0.005) and Lactobacillus group (*p* = 0.009) compared to the raw materials.

#### 3.2.4 Functional prediction analysis

**Figure 6:**
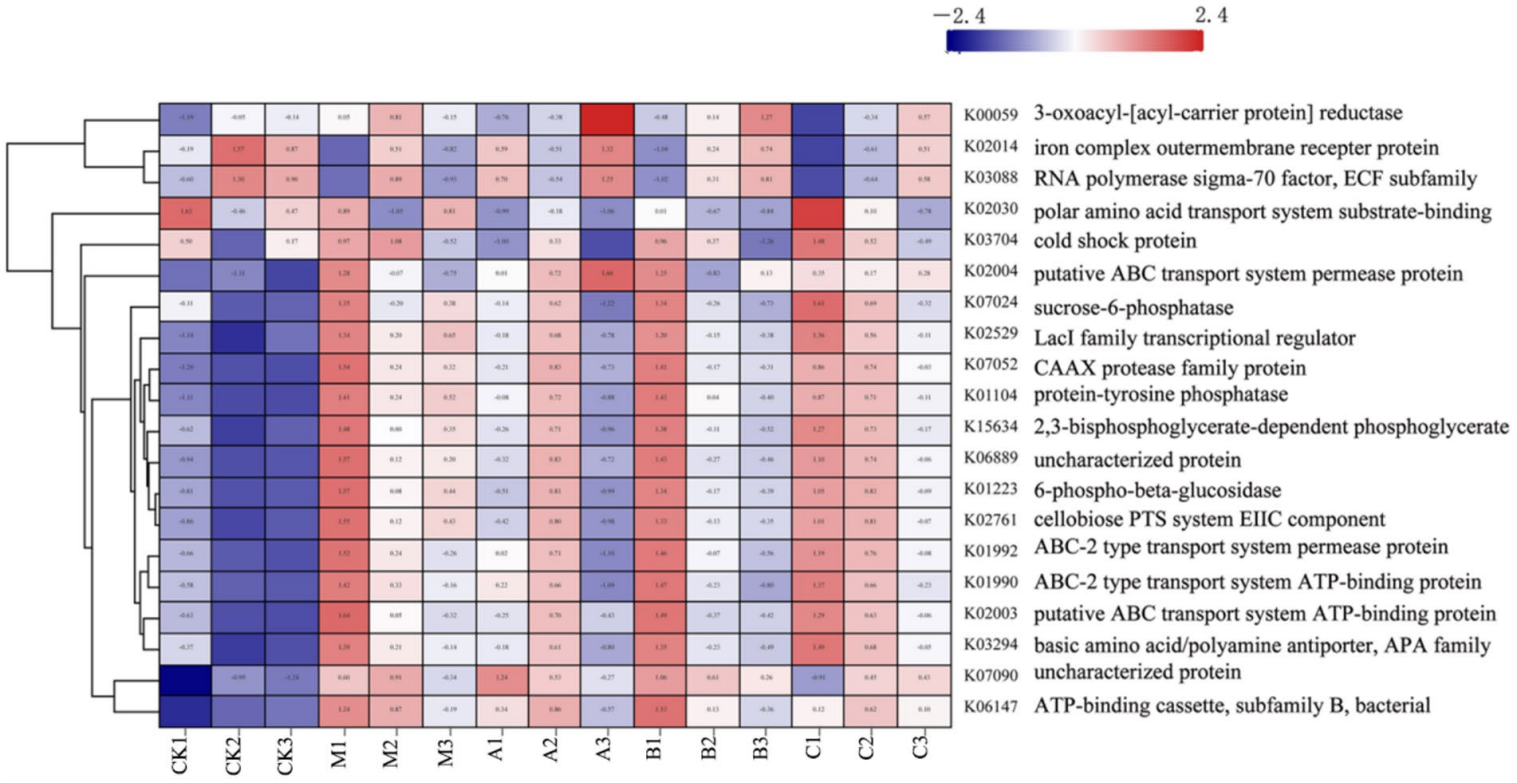
Functional clustering analysis of different additives.

**Figure 7:**
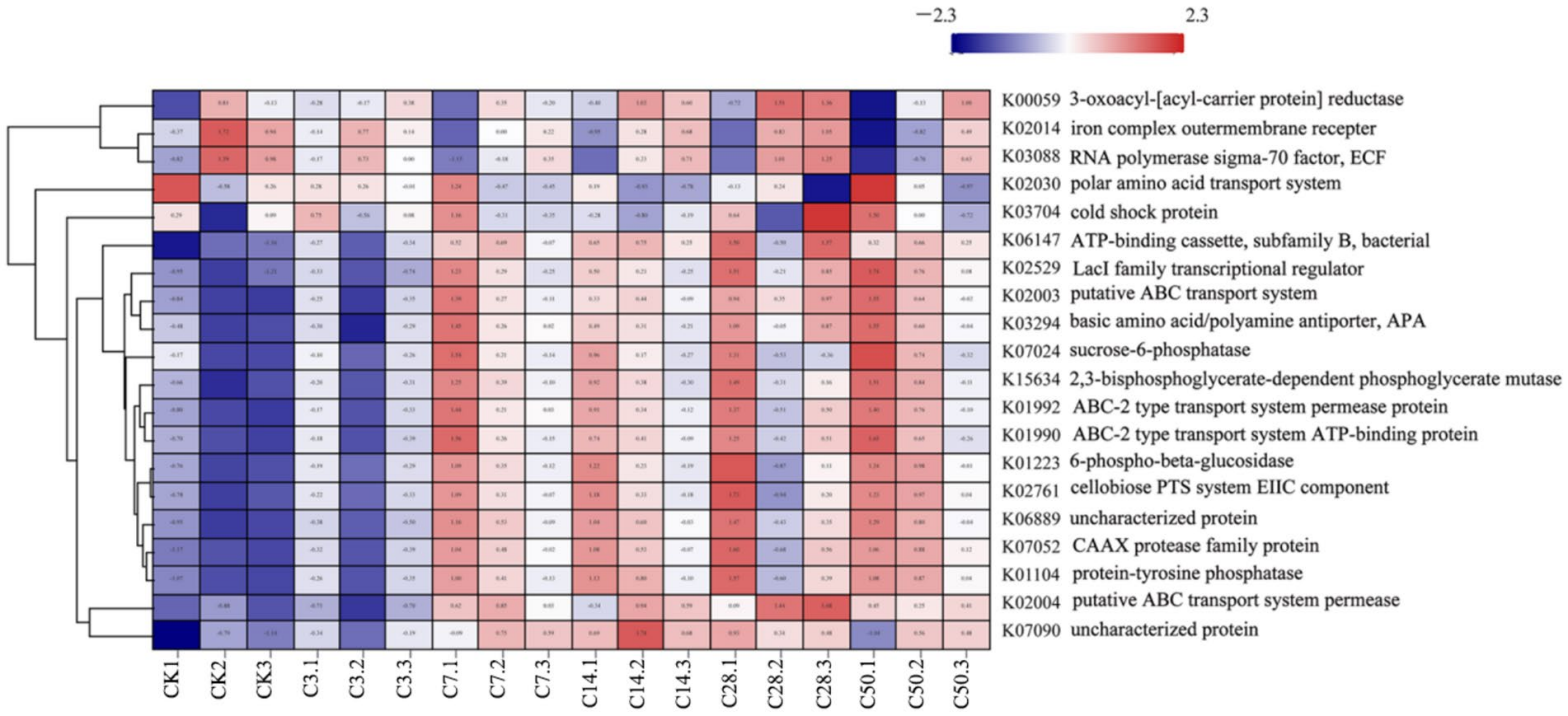
Functional clustering analysis of different silage times.

PICRUST was used for functional prediction analysis of the sample community, and relevant KEGG path information was clustered, and the cluster analysis Figures 6, 7 were drawn, and the results were as follows.

The identified functional categories comprise transporter, cytoskeleton assembly, stress resistance, and glucose metabolism.

Before silage, there was a high expression abundance of stress-resistant functions in the raw material, while processes related to cell reproduction and glucose metabolism, essential for ATP production, exhibited low expression abundance. By the third day, the functional expression of silage remained largely unchanged from the raw materials, maintaining a high level of stress resistance, with slight enhancements observed in cell metabolism and reproduction. Prolonged silage leads to a gradual decline in stress resistance expression and an increase in glucose metabolism. Although there were minimal differences among the silage groups, the overall functional abundance data varied considerably before and after silage. Comparing specific additions, we find that, except for the HMC4 experimental group’s reduced expression abundance of stress resistance, the expression abundance between the untreated blank model group and the heterozygote group is nearly identical. Both the mixed and Lactobacillus groups exhibited moderate levels of functional expression abundance, with the mixed bacteria group showing higher overall abundance than the Lactobacillus group.

## 4 Discussion

### 4.1 Nutritional value of silage

Synthesizing lactic acid and preserving feed nutrients are crucial for producing high-quality silage. When making silage from grass-based ingredients, the ideal pH of the finished product should range between 3.8 and 4.0, with a lactic acid level of approximately 6% and an acetic acid content of around 2%. This helps minimize the production of propionic acid and butyric acid (Kung et al., 2018). King grass served as the starting material for the silage experiment in this study, yielding a range of silage products differing in quality. The heterozygote group exhibited the highest lactic acid concentration at approximately 6% and a pH value of around 3.8, meeting the standards for high-quality feed. Owing to the higher concentration of Lactobacillus, such as Lactobacillus, *Candida albicans*, Lactococcus, and Weissiella, in the heterozygote group, its lactic acid concentration was significantly higher compared to other experimental groups. Furthermore, the lactic acid to acetic acid ratio in the heterozygote group was approximately 2, suggesting some Lactobacillus converted lactic acid to acetic acid in this group (Kung et al., 2018). Additionally, the model group showed a higher concentration of acetic acid. This could be attributed to the absence of additional Lactobacillus and sugars in the early stages, leading to a deficiency of Lactobacillus during the initial stages and an extended aerobic phase, thereby promoting acetic acid production (Kung et al., 2018; Zhao et al., 2019). After silage, there was a consistent decrease in crude protein concentration. Plant proteases break down proteins into biogenic amines and other compounds during the silage process. Generally, Lactobacillus inhibit plant proteases and proteolytic bacteria, leading to a rapid decrease in the surrounding environment’s pH (Dong et al., 2022). Consequently, the silage containing bacteria has a higher crude protein content compared to the blank model group, contributing to nutrient preservation. It is noteworthy that the crude protein content increased in the 28-day silage. This could be attributed to Neo-sphingomycetes in the heterozygote group breaking down biogenic amines, while the high abundance of Pantobacter partially inhibited Proteolytic bacteria (Xiang et al., 2022). The amount of soluble sugar in the feed decreased significantly due to rapid microbial growth and utilization of soluble sugars (Xiang et al., 2022). The HMC4 experimental group in this study exhibited a higher concentration of soluble sugar, possibly due to its capacity to degrade cellulose, accelerate cell wall breakdown, produce increased soluble sugars, and maintain high levels of Lactobacillus (Zheng, 2022). The study observed that the acid detergent fiber and crude fiber content in the HMC4 experimental group was significantly lower compared to the blank model group, whereas the neutral detergent fiber content remained relatively unchanged. This difference may have resulted from the higher concentration of bacteria in the experimental groups, which encouraged microorganisms to break down the fiber (Gharechahi et al., 2021).

### 4.2 Analysis of microflora in silage

Microbial activity is the primary driver of compositional changes in silage. Microbial diversity research in this study showed that the species abundance in silage remained consistent, consistent with previous findings by Chen et al. (2022). This could be attributed to the relatively consistent supply of total carbohydrates to microorganisms in the silage environment, with no additional molasses added. Although different microbial species are present in aerobic and anaerobic environments, the overall species diversity index remains constant, as verified by NMDS.

Silage serves as a vast fermentation reservoir, harboring a diverse array of thriving microorganisms. Species composition analysis in this study identified four major bacterial phyla in silage: Proteus, Bacteroides, Actinomycetes, and Thin-walled Bacteria. These findings align closely with the gastrointestinal microbial composition of Hainan black goats (Zhi et al., 2022). This may contribute to the differences in gastrointestinal microorganisms between Yimeng Black Goats and Hainan Black Goats. Additionally, this provides further evidence of the impact of nutrition on gut microbes. Numerous studies have reported the presence of amoeba, actinomycetes, and thick-walled phyla in silage products (Ali et al., 2020; Gharechahi et al., 2020; Gharechahi et al., 2021). In nature, bacteria with thick walls are among the most common microbes. The majority of Lactobacillus essential to the silage process belong to this phylum (Tian et al., 2022). Throughout the silage process, these microbes release various enzymes that break down cellulose and polysaccharides, producing organic acids (Gharechahi et al., 2021; Wang Q. D. et al., 2022). However, Proteus is considered potentially harmful (Tian et al., 2022). It facilitates sugar breakdown during the silage process, enhancing the host’s nutrient utilization (Gharechahi et al., 2021). Numerous genes linked to cellulose degradation are found in Bacteroides, which also breaks down pectin and can break down sugars quickly (Gharechahi et al., 2021; Wang Q. D. et al., 2022). When it is digested, organic acids like acetic and succinic acid are produced (Gharechahi et al., 2021). Actinomyces are crucial to preserving the ecological balance of colonies (Xiang et al., 2022).

Although thick-walled bacteria were less prevalent in this study, Lactobacillus, *Candida albicans*, and Weissiella were more abundant and played a significant role in the thick-walled bacterial community. This shift from the primary genus Streptomyces to Lactobacillus as silage duration increases enhances silage quality, consistent with the findings of Gharechahi et al. (2021). During the initial phase of silage fermentation, Lactobacillus, notably Leuconostoc and Weissella species, are pivotal in acidogenesis (Xiang et al., 2022). Conversely, during the later stages of silage fermentation, Lactobacillus dominate, a phenomenon commonly observed in various fermented products (Qiao et al., 2022; Tian et al., 2022; Xiang et al., 2022). This is because early in the fermentation process, there is a high concentration of soluble sugar, and the pH and oxygen concentration are ideal for cell growth. Weissiella and *Candida albicans* growth was inhibited in the post-fermentation stage due to the low pH values of the acidic and anaerobic environments, allowing Lactobacillus, an anaerobic species, to gradually become dominant (Wang S. R. et al., 2022). Initially in fermentation, the addition of Lactobacillus to silage rapidly breaks down soluble sugars, forming acidic compounds that further inhibit microorganisms like Weissiella (Dong et al., 2022). This study demonstrates that Weissiella abundance is reduced in conventional silage without additives; however, there is a more significant decrease in Weissiella and *Candida albicans* abundance in the Lactobacillus group. The resulting increase in Lactobacillus abundance contributes to the improvement of silage quality.

Moreover, this investigation revealed an increase in both protozoa and bacteria. Although Proteus is not beneficial for silage production, its abundance reportedly increased to nearly 40% during the silage-making process (Xiang et al., 2022). In this study, Pantobacter, Oligotrophomonas, and Neosinosine bacteria were primarily responsible for the increase in Proteus content, while the content of Acinetobacter remained largely unchanged, and the abundance of Enterobacteriaceae was suppressed. Pantobacter has been frequently reported in various fermentation processes (Xiang et al., 2022). This could be attributed to the bacterium’s high stress tolerance and its ability to thrive in acidic environments (Wang Q. D. et al., 2022). Additionally, Pantobacter had a positive effect on preserving the nutritional content of silage by inhibiting proteolysis. Oligotrophomonas is frequently utilized in agricultural production due to its ability to produce a variety of proteases and antibacterial compounds. New Sphingomycetes are commonly found in tropical crops. While its exact role remains unknown, it is believed to be one of the characteristic bacteria found in tropical silage products. It has been reported to possess metabolic capabilities, produce active compounds, and play a crucial role in the breakdown of biogenic amines, which is beneficial for silage preservation (Ali et al., 2020; Liu et al., 2021; Wang et al., 2021). Research has shown that Acinetobacter, despite being an aerobic microorganism, can thrive in anaerobic environments rich in acetic acid (Muraro et al., 2021; Chen et al., 2022; Zheng, 2022). Enterobacter is one of the main bacteria competing with Lactobacillus for soluble sugars in silage. They can degrade the quality of silage and produce endotoxins, succinic acid, and butyric acid (Wang Q. D. et al., 2022; Xiang et al., 2022). Consequently, although Proteus abundance increased in our study, the predominant increase was observed in bacteria with auxiliary functions or high stress tolerance, while the harmful bacteria were suppressed. The predominant bacteria include Flavobacterium, Sphingosine Bacilli, and Sphingomonas, among others mentioned above. Previous studies have shown that the abundance of *Pseudomonas aeruginosa* varies depending on the type of silage. For example, sorghum silage had approximately 7% while corn silage had about 3% *Pseudomonas aeruginosa* (Wu et al., 2021; Wang Y. L. et al., 2022). Based on the published data, the Lactobacillus group in this study exhibited the highest abundance (7 percent), while the heterozygous group had the lowest abundance (3.5 percent). The increase in the population of *Pseudomonas aeruginosa* following the addition of Lactobacillus may be due to its anaerobic nature (Wang Y. L. et al., 2022). *Pseudomonas aeruginosa* in the anaerobic environment in the latter stage has a material foundation thanks to the highconcentration of nutrients preserved by the quick suppression of Lactobacillus in the early stage (Dong et al., 2022). Similarly, adding Lactobacillus to the silage increased the abundance of Sphingomyces. Non-fermentative sphingosine bacilli, along with Oligotrophomonas, Flavobacterium, and Acinetobacter, contribute to the ethanol content in feed. They also produce lipase, crucial for regulating dietary crude fat levels (Ng et al., 2022). In silage, Sphingomonas can inhibit mold and yeast formation (Ali et al., 2020).

K00059, K02014, and K03088 were significantly expressed in the raw materials, as indicated by the PICRUST functional analysis. K00059 is associated with fatty acid production and bacterial motility. The transport functions of K02014 and K03088 influence the stress resistance of the strains. Gram-negative bacteria are carriers of the K02014 gene, vital for survival in low-water environments. The extracellular pressure regulator, K03088, is crucial for bacterial adaptation to water- and nutrient-scarce conditions. During silage maturation, the water content of the system proportionately increases, leading to a progressive decline in the prevalence of this function. This suggests that the moisture content of the silage environment affects the functionality of the system. Functional expressions related to cell formation and carbohydrate metabolism are also crucial. It is apparent that numerous ATP-related functional variables are significantly expressed during the silage process, indicating active energy metabolism by microorganisms during this period. The breakdown and transportation of carbohydrates by bacteria are reflected in the increased expression of K02529 and K02761. K02529 is associated with oxygen concentration, regulates the expression of genes involved in carbohydrate metabolism, and is closely linked to cell membrane mobility and cellulase secretion (Lee et al., 2017). K02761 is responsible for facilitating the transport of high-energy phosphate by the transmembrane protein EIIC into bacteria and the absorption of sugars by bacteria (Aleksandrzak-Piekarczyk et al., 2014). Additionally, K07052 is responsible for the development of the cytoskeleton and the replication of genetic material, both crucial for maintaining bacterial integrity (Pryor et al., 2013). It is evident that the expression abundances of these functional components varied before and after silage, with differences observed among the various silage additives. Particularly, the heterozygous experimental group exhibited significantly greater expression abundances of these functions compared to the other experimental groups. This illustrates how the addition of HMC4 could accelerate microbial breakdown of plant cellulose, enhance the utilization of soluble sugars, and provide energy for microbial growth. Furthermore, there was no discernible variation in biomass among the silage additive groups due to the slight alteration in total energy, consistent with the findings of the *α* analysis in the preceding phase.

## 5 Conclusion

In this study, high-quality lactic acid bacteria and cellulose-degrading bacteria were screened from the intestines of Hainan black goats. The two strains were hybridized to get heterozygotes by protoplast fusion technique. King grass silage was studied by using heterozygote and parent strain as silage additives. The results show that the addition of heterozygote can significantly increase the content of organic acid in silage products, the content of lactic acid in feed is 6.19%, the content of acetic acid is 3%, and the pH value of feed is reduced to 3.8, which belongs to high quality silage. In addition, heterozygote significantly increased the content of crude protein in silage and decreased the content of crude fiber and acid detergent fiber in silage. Compared with the mixed fermentation group, it also maintained a higher content of soluble sugar. The results showed that the addition of heterozygote could effectively maintain the nutritional value of silage.

The microbial composition of silage was studied, and it was found that thick-walled bacteria, Proteus, actinomycetes and a small number of actinomycetes were dominant in the process of king grass silage. In the process of silage, lactic acid bacteria such as Lactococcus, Weisei and *Candida albicans* were gradually replaced by anaerobic acid-resistant Lactobacillus, which became the main genus of lactic acid production in the process of king grass silage. The results of PICRUST showed that the heterozygote group had higher cellulose metabolism function, and the addition of heterozygote led to the proliferation of lactic acid bacteria in silage, which brought higher lactic acid concentration than other experimental groups.

## Data Availability

The original contributions presented in the study are publicly available. This data can be found here: (https://www.ncbi.nlm.nih.gov/sra/PRJNA1183982).
